# Providing free influenza vaccine to increase vaccination take-up and improve physical and mental health: evidence from China

**DOI:** 10.3389/fpubh.2026.1883613

**Published:** 2026-09-09

**Authors:** Yan Wang

**Affiliations:** School of Agricultural Economics and Rural Development, Renmin University of China, Beijing, China

**Keywords:** health outcome, individual welfare, influenza, public health policy, vaccination

## Abstract

**Introduction:**

Compared to developed countries, influenza vaccines have lower popularity in developing countries and are generally not given much attention. This paper analyzes the effect of China's policy of providing free influenza vaccine for the population aged 60 and above on vaccination take-up and health outcomes.

**Methods:**

Using nationwide survey data and combining differences across registered residence with differences across age cohorts included by the program. The identification strategy of difference-in-difference estimated the impact of free influenza vaccination policy on vaccination take-up, physical health, and mental health, and explore its impact mechanism.

**Results:**

This policy can increase the influenza vaccination take-up among the older population by 23% points, and yields significant improvements in both physical and mental health. Crucially, due to the time interval between vaccination and investigation, these health benefits are not driven by the vaccine's direct biological protection, but rather arise through indirect behavioral and belief pathways. Specifically, the policy reduces the probability of reporting that health limits daily activities by 16.9% points, and increases the likelihood of feeling energetic by 28.1% points.

**Discussion:**

The findings of this article provide experience for promoting influenza vaccine in developing countries and discover the indirect physical and mental health improvements that influenza vaccination may bring.

## 1 Introduction

The spread of viruses is a serious public health issue, that not only affects individuals' health but also has a huge impact on the health sector and the overall economic operation (1, 2). Vaccination as the most effective instrument used for virus protection has been widely recognized, and the results of the estimation indicate that vaccination can reduce millions of deaths from preventable diseases every year (3, 4). For the influenza virus, there are approximately 1 billion cases of infection each year, including 3 to 5 million severe cases and approximately 290,000–650,000 deaths from respiratory diseases caused by the influenza virus (5).

The utilization of influenza vaccines has significant regional differences, especially in developing countries and low-income countries. Due to the difficulty in obtaining influenza vaccines and the lack of corresponding vaccine policies, people in these regions face challenges in obtaining the protective benefits of vaccination^1^. Consequently, efforts to improve health outcomes in developing countries through the widespread dissemination of vaccines are highly significant. Furthermore, the reliable identification and assessment of the effect of vaccination policies on vaccination rates and health outcomes are crucial. Such evaluations can provide robust support for decision-making within the health sector.

This article leverages the introduction of the free influenza vaccination policy targeting registered residents aged 60 and above in Beijing and Shanghai to study its effects. Using individual-level data from the Chinese General Social Survey (CGSS) and a difference-in-differences framework that exploits age and residence-based eligibility, I examine the direct impact of the policy on vaccination take-up, spillover effects within households, and improvements in physical and mental health through detailed survey questionnaire data, and explored relevant mechanisms. This study not only contributes to further improving vaccination coverage and providing immune protection for the older population, but also helps to understand the medium-term indirect health improvements through vaccination.

This paper is situated within three distinct strands of literature. The first strand concerns the determinants of vaccination take-up, including information dissemination (7–10), risk perception (11–13), mandatory policies (14–16), and personal hesitation toward vaccines (17–19). Within this context, this paper specifically investigates the role of offering free vaccination as an incentive. While existing research consistently confirms that free vaccines effectively increase vaccination rates (20–24), and that additional cash or non-cash rewards are also effective (25–27). This paper provides new evidence by exploiting regional and age-based eligibility to evaluate how such policies affect both vaccination behavior and health outcomes.

The second strand to which this paper contributes is the literature on free influenza vaccination in developing countries. Prior evidence, such as Bouckaert et al. (20) and Brilli et al. (21), has focused exclusively on developed countries like the Netherlands and Italy, showing significant effects among the older population. However, given that influenza vaccination rates are generally much lower in developing countries–and it remains unclear whether this is due to high prices or low awareness–the applicability of developed county findings is questionable. To my knowledge, this paper provides the first empirical evidence in the impact of free influenza vaccination policy on vaccination take-up and health outcomes in developing country setting.

The third strand to which this paper contributes concerns the health effects of free vaccination policy and their underlying mechanisms, offering actionable policy implications for improving national health in developing countries. Regarding physical health, the literatures have reached a consistent conclusion: large-scale vaccination campaigns can save lives, and reduce the probability of disability and mortality (2, 28–31). However, the impact on mental health remains a subject of ongoing debate. On one hand, Baranov, Bennett and Kohler (32) find that providing free Antiretroviral Therapy (ART) to fight HIV in Malawi can improve the mental health of recipients. Similarly, during the COVID-19 pandemic, Coley et al. (33) and Chaudhuri and Howley (34) document that vaccination effectively reduces depression. On the other hand, Awijen et al. (35), using data from 194 countries, find that the introduction of the COVID-19 vaccine increased online searches for pessimistic words, suggesting that vaccine availability may have induced fear and anxiety among local populations. By evaluating both physical and mental health outcomes, this paper helps to reconcile these conflicting views within the context of developing country.

## 2 Institution background and research hypothesis

### 2.1 Vaccination policy in China

In China, vaccination is implemented under the unified management of the government. According to China's laws and regulations, vaccines are divided into two categories. The first category is vaccines within the National Immunization Program (NIP)^2^. Residents living in China have the right and fulfill the obligation to receive vaccines under the NIP by the law, and the vaccines within the NIP are provided free of charge by the government. The second category is vaccines without the NIP, which require residents voluntarily at their own expense to receive. There are differences in voluntariness and welfare between two different types of vaccines.

### 2.2 Influenza vaccination in China

In China, influenza vaccines are not in the NIP, residents need to pay for themselves to receive. Beijing has been using influenza vaccines since 1996, and after more than 10 years of observation, it has been proven that influenza vaccines can provide good protection. Therefore, in 2007, the government of Beijing officially provided free influenza vaccines for population aged 60 and above^3^. In the first year of implementation, 2.02 million older adults in the city eligible for vaccination, 170,000 older adults over 60 years old were vaccinated free of charge against influenza in 4 days^4^ Similarly, in 2009, Shanghai provided free influenza vaccines to population aged 60 and above in response to the H1N1 influenza virus and to protect the health of vulnerable groups^5^

### 2.3 Research hypothesis

Due to the implementation of the free influenza vaccination policy, the influenza vaccine that originally required self-payment can be obtained for free by eligible citizen, relaxing budget constraints. This generates the first research hypothesis of this article:

**H1**. *The free influenza vaccination policy helps to increase the influenza vaccination coverage among older population*.

As this article focuses on the medium-term health improvement effects of the vaccination policy on affected populations, eligibility for vaccination may not directly improve health. However, considering that additional health or policy information may be obtained during the vaccination process, which may lead to changes in health behavior and beliefs, this change may improve health outcomes in the long term. This leads to the second research hypothesis and mechanism hypothesis of this article:

**H2**. *The free influenza vaccination policy can improve the medium-term health outcomes of older population*.
**H3**. *The free influenza vaccination policy mainly improves the physical and mental health of the older population by changing their health behaviors and beliefs*.

## 3 Data and identification strategy

### 3.1 Data source and sample restrictions

The data used in this study is from the 2010 wave of CGSS. Although CGSS has years of survey data, it only includes the health module of the East Asian Social Survey in the 2010 and 2021 waves, which involves issues related to influenza vaccination and detailed inquiries about the health status and behaviors of respondents. However, due to the ongoing COVID-19 pandemic in 2021, there may be systematic biases in the estimated results. Therefore, the baseline results of my analyze are only using the 2010 wave, and additional robustness tests are conducted using the 2021 wave. The data includes a total of 11,783 observations, from which I obtained two samples.

Each sample includes records of personal basic information, vaccination status, health conditions, and health behaviors. I consider the first sample based on individual age, which included individuals aged 51–70^6^, to estimate the direct and health effects of the eligibility for free influenza vaccination (called as main sample). Due to the inclusion of family member information reported by respondents in the data, this information can be used to determine the vaccination eligibility of spouse and estimate the spillover effects. Subsequently, I screen the second sample based on the age of the spouse, selecting individuals who are married and whose spouse is between 51–70 years old. However, I remove individuals who are of the same age as their spouse and did not consider situations where both the individual and spouse are eligible or ineligible at the same time (called as spouse sample).

According to the research of Bouckaert et al. (20), vaccination has spillover effects within the family that could violate the Stable Unit Treatment Value Assumption (SUTVA). Although their results indicate that such spillover effects do not flow from older spouse to younger one, my discussion in Section 4.3 demonstrates the presence of these spillover effects in my sample. Consequently, in my main sample, I exclude individuals from Beijing and Shanghai who are aged 60 and below and have spouse more than 60 years old, to ensure that SUTVA is met.

All two samples are restricted to individuals (the individual themselves or their spouse) aged between 51 and 70, with observations containing missing values for the primary dependent and control variables excluded^7^. The remaining 1,224 observations are used to estimate the direct effects on individuals, 609 observations are used to estimate the spillover effects between spouse.

### 3.2 Variable description and summary statistics

The primary outcome variable in the analysis is the influenza vaccination take-up. As a basic outcome, I define the self-reported vaccination status in the 2009 influenza season as an indicator of vaccination take-up. In the subsequent sections, I extend the scope of analysis to health outcomes and health behaviors, measured through a range of subjective or objective indicators^8^.

According to the policy, the eligibility criteria for free vaccination are based on specific birth dates; people aged 60 and above in 2009 (born before December 31, 1949) are eligible for free influenza vaccination during the corresponding influenza season. Therefore, I redefine individual ages by calculating the difference between the questionnaire year and the year of birth to avoid errors arising from discrepancies between the survey date and specific birth dates, ensuring the accurate determination of eligibility. After recalculating, people aged 61 and above in 2010 in Beijing and Shanghai met the policy criteria for free influenza vaccination.

I include several covariates in my analysis. Covariates are individual demographic characteristics, including age, gender, education level, marital status, ethnicity, and other information. Table 1 presents the descriptive statistical results of the main sample, where the first column shows the sample mean and the second column shows the standard deviation. Only 6% of the people aged 51–70 in China have received the influenza vaccine, far below the vaccination rate target set by the WHO. China has implemented a series of basic medical insurance reforms since 1998, resulting in a coverage rate of over 90% for basic medical insurance. However, even so, the poor health status of older people in China is still difficult to solve, with 53% of them reporting chronic diseases.

**Table 1:** Descriptive statistics.

Variable	Mean	Standard deviations
Influenza vaccination	0.06	0.24
Age	59.07	5.38
Male	0.54	0.50
**Education level**		
Primary	0.54	0.50
Lower secondary	0.25	0.44
Upper secondary	0.14	0.35
Post secondary	0.06	0.24
Nation: Han	0.91	0.29
Medical insurance coverage	0.92	0.28
**Household income**		
< 15K	0.28	0.45
15K−35K	0.32	0.47
>=35K	0.29	0.45
Missing	0.12	0.32
Suffering from chronic diseases	0.53	0.50
Married	0.88	0.32
**Number of children**		
0	0.01	0.11
1	0.26	0.44
2	0.31	0.46
3	0.24	0.43
4+	0.17	0.38
Observation	1,224	

All mean and standard deviation show weighted sample. The descriptive statistics of the spouse sample, other outcome variables, and alternative explanatory variables used in this paper are presented in Supplementary Tables A1, A2.

### 3.3 Identification strategy

The individual's age and registered residence co-determine the eligibility for free influenza vaccination. In order to estimate the effect of the policy on the older population, I follow the method of Duflo (36) and estimated a cross-sectional difference-in-difference (DID) model. All regression clustering at the city level to address individual-related issues, and employ survey weight for weighting purposes for cross-sections to correct for possible sampling bias and the limited sample size of singles. The specific form is as follows:


$$\begin{array}{l} Y_{isy}=\alpha \;+\;\beta Treat_{s}\;\times agePost_{iy}\;+\gamma X_{i}\;+\theta_{s}\;+\eta_{y}\;+\epsilon_{isy} \end{array}$$ (1)

Where *Y*_*isy*_ is the outcome variable of individual *i* with age *y* in region *s*, *Treat*_*s*_ is a dummy variable for the policy in region s, set 1 for Beijing and Shanghai, and 0 for other regions. *agePost*_*iy*_ is a dummy indicating whether individual *i* of age *y* meets policy requirements, set 1 for aged 61 and above, and 0 for others. *X*_*i*_ is a series of individual and family control variables, η_*y*_ is the fixed effect of age, θ_*s*_ is the fixed effect of region, and ϵ_*isy*_ is the residual term. Among them, β is the DID estimation coefficient that I am interested in.

The basic idea behind the identification strategy can be illustrated using simple two-by-two tables. Supplementary Table A3 presents this result, showing the mean and difference in vaccination rate for different regions and age cohorts. Intuitively demonstrating the direct effect of the policy on vaccination rate.

## 4 Result

### 4.1 Baseline result

The first step in my empirical analysis is to determine the effect of the policy on the vaccination take-up for older population. The identification strategy can be extended to a regression framework, using Equation 1 to estimate the causal effect.

Column 1 of Table 2 shows the estimated results of Equation 1. The results indicate that the policy will lead to a 23% points increase in the vaccination take-up. Compared to the average of the control group, the average vaccination rate increased by 338% from 6.8% points. My conclusion is consistent with existing research that providing free influenza vaccination to the older population can effectively improve vaccination take-up. However, my estimation results significantly differ in the magnitude of the effect compared to the evidence obtained from developed countries. Brilli et al. (21) report that Providing free influenza vaccine for people aged 65 and above in Italy will lead to 7.9% points increase in influenza vaccination take-up (average of control group 23.4% points, an increase of 34%), while Bouckaert et al. (20) based on the Netherlands reported result is 9.8% points (average of control group 40% points, an increase of 25%). In contrast, the policy in China has a greater impact on the vaccination take-up, which is more than twice of developed countries. In addition, due to the relatively low baseline vaccination rate for basic vaccines in China, the relative growth rate appears much higher than that of developed countries. However, it should be noted that this substantial relative increase does not necessarily reflect the effectiveness of policy interventions; rather, it may simply be a mathematical amplification effect arising from the low starting point.

**Table 2:** Effect of the free influenza vaccination on vaccination take-up.

Dependent variable	Vaccination take-up					
	(1)	(2)	(3)	(4)	(5)	(6)
Over 60 years old × Beijing or Shanghai	0.229^***^ (0.082)	−0.021 (0.029)	0.231^**^ (0.089)	0.235^***^ (0.081)	0.227^***^ (0.053)	0.236^***^ (0.059)
Over 60 years old × Per capital number of medical and health institutions			−0.009 (0.022)			−0.011 (0.024)
Over 60 years old × Local incidence rate				0.004 (0.006)		0.005 (0.006)
Over 60 years old × Working status					−0.059 (0.043)	−0.066 (0.044)
Beijing or Shanghai × Working status					−0.022 (0.094)	−0.023 (0.094)
Mean of control group	0.0677	0.117	0.0677	0.0677	0.0677	0.0677
Province FE	√	√	√	√	√	√
Age FE	√	√	√	√	√	√
Observations	1,224	1,645	1,224	1,224	1,224	1,224

All regressions control individual attributes. All regressions employ survey weight for weighting purposes. The SEs reported in parentheses is clustered at the city level. Significance level: ^***^p < 0.01; ^**^p < 0.05; ^*^p < 0.1.

### 4.2 Robustness test

#### 4.2.1 Placebo test

I conduct a control experiment and select the group aged 41–60 from the original sample, reset *agePost*_*iy*_, and set the group aged 51–60 to 1 and the group aged 41–50 to 0 in the sample, finally re-estimate Equation 1. If there are systematic differences in vaccination take-up in different regions, people in regions implementing the free vaccination policy have higher vaccination take-up with increasing age, then the results will show a positive coefficient, indicating that the effect obtained from my basic results does not come from the free vaccination policy, but from the differences in growth patterns between regions. The results are shown in column 2 of Table 2, indicating that the impact is minimal and not statistically significant. Although this is not definitive evidence, it can still indirectly demonstrate the effectiveness of the assumptions required for my identification strategy.

In addition, to demonstrate that my results are reliable and not due to accidental factors, I also conduct another placebo test. I randomly assigned the policy implementation indicator to different regions and re-estimated Equation 1 to obtain new estimation results. This process repeats 1,000 times to observe the distribution of the estimation outcomes, which are compared with the results presented in Table 2. The results indicate that the mean of the estimates obtained from the randomly assigned policy implementation regions is approximately equal to zero, showing a significant difference from the estimates derived from the actual policy, demonstrates that the policy effects are not due to chance. The specific results are shown in Supplementary Figure B1.

#### 4.2.2 Alternative interpretations

The baseline results have demonstrated the impact of the free influenza vaccination policy on vaccination take-up. But one may argue that the increase in vaccination take-up is not caused by the policy, but by other related competitive factors. Here, this article further discusses the impact of other possible confounding factors on the results to demonstrate the robustness of the baseline results.

This article considers the following three competitive confounding factors, which are incorporated into the equation and compared with the coefficient results of interest. Firstly, considering that changes in healthcare infrastructure construction may have differentiated impacts on different age groups (37, 38), I collect the per capita number of medical and health institutions data in various provinces from the National Bureau of Statistics of China in the year before vaccination. The interaction term between the per capita number of medical and health institutions and the dummy variable of age cohort is included in the equation, and the results are shown in column 3 of Table 2. Second, considering that regional disease risk differences may have a differentiated impact on different age groups (12, 13), I collect provincial influenza incidence rate data in the year before the influenza season from the Chinese Center for Disease Control and Prevention and add the interaction between influenza incidence rate and treatment cohort dummy variables in the equation. The results are shown in column 4 of Table 2. Thirdly, due to the mandatory retirement age stipulated by China's law, it may coincide with the vaccination age mentioned in this article, leading to retirement becoming an important confounding factor (39–41). I use the information on whether the respondents were working in the questionnaire, add it to the equation, interacting with age cohorts and regional groups to eliminate the impact of retirement. The results are shown in column 5 of Table 2. Finally, column 6 of Table 2 also shows the results of incorporating all alternative interpretations into the specification.

#### 4.2.3 Measurement error in registered residence

It is worth noting that all of my estimation results so far are based on the location of the respondents, rather than the required registered residence by policy. The main reason is that the CGSS data is only limited to the county-level administrative regions to the inquiry of the location of the respondents' registered residence, and it is impossible to further determine whether them belong to the province-level or municipality-level administrative regions. To protect the privacy of the respondents, CGSS data also does not disclose detailed information about the location of registered residence, so I use the location of the respondents to replace the location of registered residence. Such operations may lead to the division of people whose registered residence is not in Beijing or Shanghai into Beijing or Shanghai, and the wrong judgment of individuals who are not eligible for the free influenza vaccination as eligible, leading to an underestimated result. However, underestimated results do not mean they are meaningless. My results indicate that even if underestimated, the effects of the policy are still significant, and the real effects will only be higher than my results, and my results are only a lower bound. In addition, since I limit the sample to aged 51–70, the older population with no registered will face higher costs when migrating to Beijing or Shanghai, so the individuals that cannot be judged in the data are more likely to come from other areas of Beijing or Shanghai, and also has registered residence, so the impact on the results may be small^9^. Finally, I delete those individuals in the sample who are in Beijing or Shanghai but cannot determine the location of their registered residence. The reduction of the sample will lead to inaccurate estimates, but similar results can also be obtained, which are shown in Supplementary Table A5.

My analysis only used survey data from the 2010 wave, and the sample size is limited, which may be subject to chance. To prove that my conclusion is not accidental, I further use CGSS survey data from the 2021 wave as a basis and obtain new estimation results using the same processing method, which is presented in the Supplementary Table A6. The results indicate that during the 2020 influenza season, the policy can still increase the vaccination take-up of the target population by 13% points, which is similar to my baseline results, but smaller than those obtained from 2010 survey data. This is mainly due to the COVID-19 pandemic during the 2020 influenza season, the potential risk of infection will be a part of the cost of suppressing the effectiveness of the free policy (25). These conclusions not only demonstrate the robustness of my findings but also validate that sudden public health problems can suppress existing public health policy, providing the experience for policy-making during crisis periods.

#### 4.2.4 Parallel trend test

My identification strategy is based on the parallel-trend assumption: for the affected population, in the absence of the policy, there should be no regional differences in the trend of vaccination take-up. Although the counterfactual is certainly unobservable, I generalize the basic identification strategy to provide evidence indirectly to support the parallel-trend assumption. Here I consider the following equation to estimate the effect of the policy:


$$\begin{array}{l} Y_{isy}=\alpha +\overset{70}{\underset{\begin{array}{c} k=51\\ k\neq 60 \end{array}}{\sum }}\beta_{k}Treat_{s}\times age_{ik}\;+\gamma X_{i}\;+\theta_{s}\;+\eta_{y}\;+\epsilon_{isy} \end{array}$$ (2)

where *age*_*ik*_ is a dummy variable of whether individual *i* age is *k* in 2010, with k ranging from 51 to 70 and 60 as the control. β_*k*_ is the estimated coefficient which I am interested in, and the definition of all other variables is the same as Equation 1.

**Figure 1:**
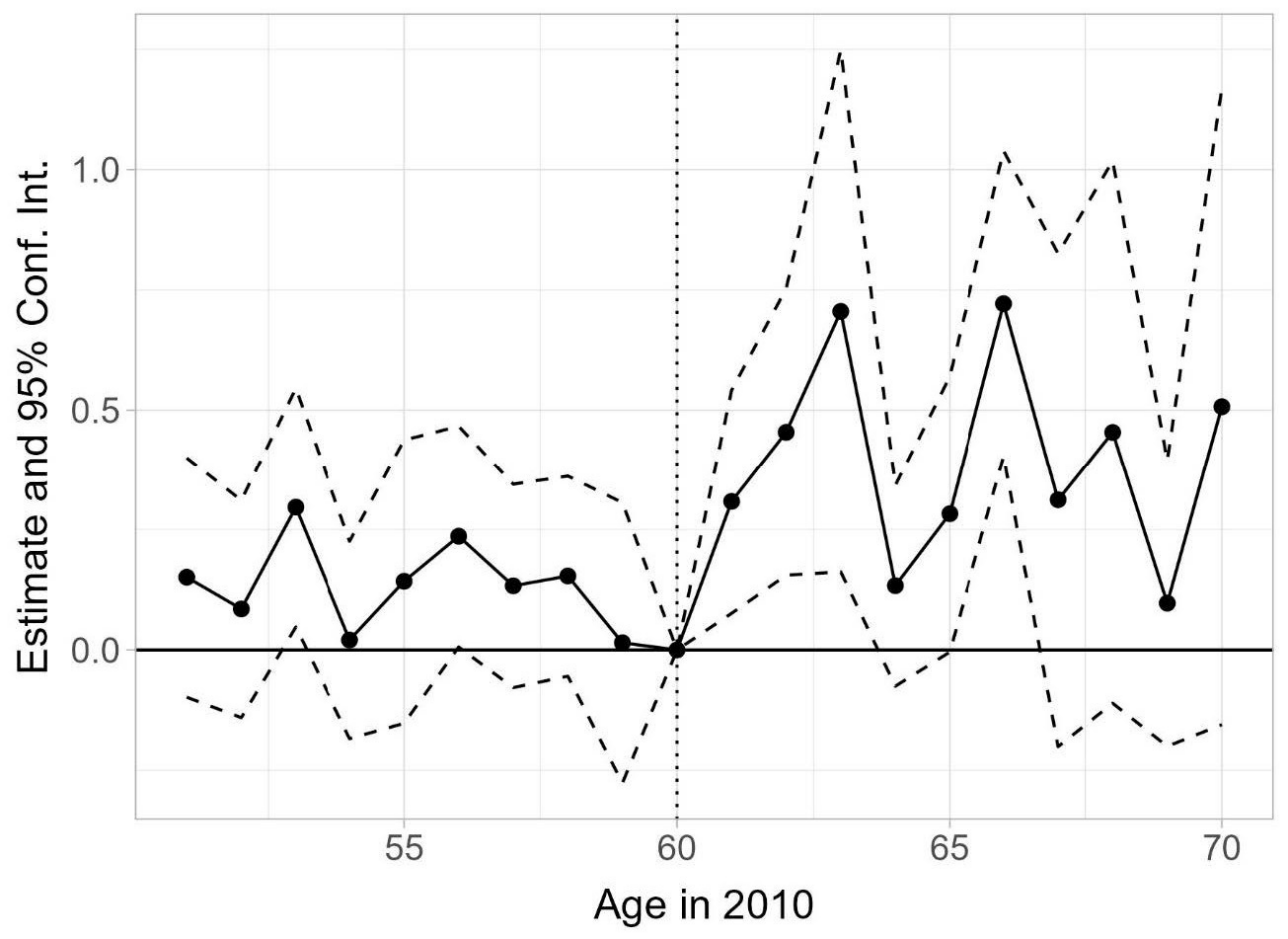
Effect of the free influenza vaccination on different age. Supplementary Table A7 presents these results in table format.

Figure 1 plots the β_*k*_, each points represent the interaction coefficient between different age and regions (95% confidence interval plotted by dashed lines). Until the age of 60, these coefficients fluctuate around 0 and cannot be statistically distinguished from 0, after which there is a significant increase. As expected, this policy has almost no impact on the vaccination take-up of groups who are not eligible for free vaccination but has a positive impact for eligible groups. The impact is mainly concentrated among younger individuals in the population aged 61–70, while the impact on older age groups is still mostly positive, but estimates have become inaccurate. There is no evidence to suggest a systematic trend for the population below 60 years old, which satisfies the parallel-trend assumption required for identification.

Although the results estimated by Equation 2 indicate that there is no systematic trend difference among the population below 60 years old in different regions, I still have concerns about the satisfaction of the parallel-trend assumption required for estimation. To address this issue, I further validate my results using the generalized synthetic control method (42), which is an improvement on the synthetic control method (43, 44). Using multiple control units to construct a synthetic control group that is analogous to the treatment group, serving as a counterfactual. This approach can effectively mitigate the issue of unreliable estimates in the DID method due to the violation of parallel trend. The results are presented in the Supplementary Tables A8, A9, which are similar to the existing results.

### 4.3 Spillover effect in the family

Due to the possible spillover of free vaccination within the family, it may lead to changes in the vaccination behavior of other family members. Here, I use a sample of married individuals with spouse aged between 51–70 to study the spillover effects of the policy among spouse. Table 3 presents the results, where column (1) estimates the effect of the spouse's eligibility for free influenza vaccination on the individual's vaccination. The results indicate that the spouse's eligibility for free influenza vaccination does not affect the individual's vaccination.

**Table 3:** Spillover effect of the free influenza vaccination within family.

Dependent variable	Vaccination take-up		
	(1)	(2)	(3)
Spouse over 60 years old × Beijing or Shanghai	−0.005 (0.117)	0.074 (0.148)	−0.180^***^ (0.068)
Mean of control group	0.0843	0.304	0.111
Province FE	√	√	√
Age FE	√	√	√
Observations	609	235	374

Significance level: ^***^p < 0.01; ^**^p < 0.05; ^*^p < 0.1.

Furthermore, I consider different scenarios of vaccination spillover between spouse. I divide the original spouse sample into two sub-samples: one with a spouse younger than the individual and the individual being eligible for free vaccination, and the other with a spouse older than the individual and the individual not being eligible for free vaccination. I re-estimate the spillover effects. The results are presented in the columns (2) and (3) of Table 3, respectively. The results indicate that when the spouse is younger than the individual and the individual is eligible for free vaccination, the impact of the spouse's free influenza vaccination on the individual's influenza vaccination is not significant. However, when the spouse is older than the individual and the individual does not have the eligibility for free vaccination, the spouse's eligibility for free vaccination will have a negative impact, resulting in an 18.7% points decrease in the probability of the individual receiving the influenza vaccination.

My results are both consistent and different from the conclusions drawn from existing studies on vaccination spillover within the family. The similarity lies in the fact that having a young spouse eligible for free vaccination does not encourage the older spouse to take up the vaccine. The main reason is that the individual already has the eligibility for free vaccination, so the spouse's eligibility for free vaccination does not affect their vaccination decisions. The difference is the impact on the vaccination of younger spouse when older spouse is eligible for free vaccination. The results of Bouckaert et al. (20) indicate that when older spouse is eligible for free vaccination, it can lead to younger spouse being vaccinated together. For such results, Fadlon and Nielsen (45) propose a theory suggests that after experiencing the impact of health shocks, family members will become aware of health information and immediately improve their health behaviors. Therefore, after individuals are eligible for free policy and take up the vaccination, family members can also learn about vaccination information, thereby changing their health behavior. My results validate another theory: when individuals realize that their probability of being infected largely depends on the vaccination behavior of other individuals they frequently come into contact with, they will adjust their vaccination strategies based on the vaccination behavior of others, resulting in a free-riding phenomenon (46). Therefore, when the spouse is covered by the free policy and takes up the vaccination, the individual will stop the vaccination behavior.

The possible reason for such differences lies in the income gap between developed and developing countries, which leads to different marginal effects of currency, further influencing people's vaccination decisions. In developed countries, due to the generally higher income of people, the marginal effect of currency is lower, and they are more concern about the effectiveness of vaccination, rather than price. However, in developing countries, generally lower incomes lead to a high marginal effect of currency, and even though people are aware of the benefits of vaccination, the marginal effect of vaccination cannot exceed the marginal effect of currency, resulting in the observed results. In addition, the results in Table 2 also indirectly support this theory. Although the number of medical and health institutions, disease incidence rate, retirement, and other factors may affect vaccination, in my conclusion, for influenza vaccination, these factors have no significant impact, price appears to be an important driver, since these observable confounders don't explain the pattern^10^.

### 4.4 The heterogeneous effects of policy

There is strong heterogeneity effect of the policy on vaccination. For different genders, the basic vaccination rate for female is lower than male, but the impact of free vaccination eligibility on female is twice that male. For different health status, the eligibility can only impact individuals with chronic disease. For different education level, the policy has a huge effect on the group with education levels of primary school and below, but the impact on other groups is relatively small. Finally, the policy has a huge impact on the group with frequency of lower media usage but higher social activity, but the impact on the other group is not significant. All results are presented in Supplementary Figure B3.

## 5 Further analyzes

I have documented the direct impact of the free influenza vaccination policy on vaccination take-up and the spillover effects within households. In this section, I further investigate the policy's impact on improving health outcomes and the possible mechanism, in order to evaluate this policy more comprehensively.

### 5.1 The health effects of the vaccination policy

There is increasing evidence from worldwide that efforts aimed at improving health can achieve the expected results. Here, I further evaluate the health effects of the policy through Equation 1. Firstly, vaccination policy can impact on physical health. The influenza vaccine is administered between September and November each year, with antibody levels reaching their peak 1 month after vaccination and beginning to decline 3 months later. The peak period of influenza incidence is mainly concentrated between December and February of the following year, the influenza vaccine effectively protects against the disease. The CGSS data questionnaire used in this paper was collected from June to August of the second year, which is no longer the peak period of influenza incidence. The survey measures the health status of respondents by asking them about their past 4 weeks. Due to the temporal differences, the health status at the time of investigation is minimally affected by influenza virus infection. My results should be interpreted more as the indirect, medium-term health improvement effect of influenza vaccination on health outcomes.

I use multiple methods to measure health outcomes, the results estimated using Equation 1 are shown in columns 1–5 of Table 4. The results show that the medium-term health improvement effect brought by the eligibility for free vaccination is significant, reflected in daily life and perceived. Increase the probability of self-reported health by 9.7% points and reduce the probability of daily life being affected by health issues by 9–17% points. But it does not have an impact on restricting climbing stairs.

**Table 4:** Effect of the free influenza vaccination on physical health.

Dependent variable	Self-evaluation of health	Health issues restrict climbing stairs	Health issues restrict the ability to move tables, use vacuum cleaners, play bowling or golf	Affect daily activities due to health	Inability to complete expected work due to health
	(1)	(2)	(3)	(4)	(5)
Over 60 years old × Beijing or Shanghai	0.097^***^ (0.035)	0.021 (0.045)	−0.092^**^ (0.042)	−0.169^***^ (0.052)	−0.136^***^ (0.039)
Mean of control group	0.425	0.256	0.215	0.204	0.177
Province FE	√	√	√	√	√
Age FE	√	√	√	√	√
Observations	1,223	1,218	1,223	1,220	1,223

All regressions control individual attributes, consider all alternative interpretations, employ survey weight for weighting purposes. The SEs reported in parentheses is clustered at the city level (Same in subsequent tables.). Significance level: ^***^p < 0.01; ^**^p < 0.05; ^*^p < 0.1.

Secondly, I also use different methods to measure subjective and objective mental health outcomes. Columns 1–5 of Table 5 show the results estimated using Equation 1. The results show that eligibility for free vaccination can lead to various improvements in mental health. Significantly reducing the frequency of pessimistic emotions and bringing positive feelings, also reduces the probability of emotional problems affecting daily life by 10–11% points.

**Table 5:** Effect of the free influenza vaccination on mental health.

Dependent variable	Feel depressed or discouraged	Feel calm and composed	Feel energetic	Unable to work due to emotions	Feel distracted due to emotions
	(1)	(2)	(3)	(4)	(5)
Over 60 years old × Beijing or Shanghai	−0.118^***^ (0.042)	0.222^***^ (0.044)	0.281^**^ (0.111)	−0.099^**^ (0.039)	−0.107^**^ (0.044)
Mean of control group	0.288	0.762	0.615	0.155	0.202
Province FE	√	√	√	√	√
Age FE	√	√	√	√	√
Observations	1,220	1,223	1,222	1,223	1,222

Significance level: ^***^p < 0.01; ^**^p < 0.05; ^*^p < 0.1.

### 5.2 Mechanism analysis

The change in vaccination behavior caused by the eligibility for free influenza vaccination may also affect people's other health behaviors, thereby improving their physical health. Table 6 presents the estimated effect on health behaviors. The results indicate that eligibility for free vaccination has no significant impact on the frequency of addictive behaviors, including smoking and drinking. But it can increase people's frequency of seeking medical treatment, increasing the probability of at least one visit per month by 15.6% points. The increase in the frequency of medical service usage can be explained by the satisfaction of medical demand, and the eligibility for free vaccination reduces the probability of older population sick but intentionally not visit doctor^11^.

**Table 6:** Effect of the free influenza vaccination on health behaviors.

Dependent variable	Smoking multiple times within a month	Drinking multiple times within a month	Visit hospital at least monthly	Accept traditional Chinese medicine treatment	Sick but intentionally not visit doctor
	(1)	(2)	(3)	(4)	(5)
Over 60 years old × Beijing or Shanghai	−0.060 (0.038)	−0.001 (0.035)	0.156^***^ (0.032)	0.206^***^ (0.043)	−0.219^*^ (0.123)
Mean of control group	0.395	0.313	0.175	0.304	0.509
Province FE	√	√	√	√	√
Age FE	√	√	√	√	√
Observations	1,224	1,222	1,223	1,224	1,115

Significance level: ^***^p < 0.01; ^**^p < 0.05; ^*^p < 0.1.

Next, I consider the eligibility for free vaccination as a way to improve mental health by changing people's belief. The results are shown in Table 7. Indicating that people who are eligible for free influenza vaccination have an increased probability of believing in social equity and life happiness by 12.7% and 25.1% points, respectively. This change in beliefs will further lead to improvements in mental health (48). On the other hand, the eligibility for free vaccination can also reduce people's anxiety about healthcare. Although the eligibility does not significantly reduce people's concerns about needing medical services but not being able to obtain, it significantly reduces concerns about being unable to pay for medical expenses due to serious illness by 27.6% points. This is very reasonable because the policy only exempts the fees and does not reduce the threshold for obtaining, thus only reducing people's concerns about medical expenses. It is precisely because of this reduced concern about medical expenses that people's mental health has improved (49, 50).

**Table 7:** Effect of the free influenza vaccination on beliefs.

Dependent variable	Believe that social is equity	Believe that life is happy	Worry about obtaining medical services	Worry about medical expenses
	(1)	(2)	(3)	(4)
Over 60 years old × Beijing or Shanghai	0.127^**^ (0.062)	0.251^***^ (0.090)	−0.046 (0.094)	−0.276^***^ (0.046)
Mean of control group	0.231	0.661	0.671	0.78
Province FE	√	√	√	√
Age FE	√	√	√	√
Observations	1,219	1,222	1,219	1,216

Significance level: ^***^p < 0.01; ^**^p < 0.05; ^*^p < 0.1.

## 6 Discussion

This study comprehensive analysis the impact of China's free influenza vaccination policy on vaccination take-up and health outcomes. Using nationwide CGSS data and evaluating causal effects by the DID method. The findings provide new evidence for the development and improvement of public health policy related to influenza vaccination.

The results indicate that the free influenza vaccination policy can significantly improve the vaccination of the older population, but it will also further curb the vaccination behavior of spouses. In terms of health outcomes, vaccination eligibility can improve the physical and mental health of older population, mainly through changing health behaviors and beliefs.

Notably, the policy of providing free influenza vaccines for the older population in China, as presented in this article, is generally larger compared to existing research based on developed countries. The main reason is that as a developing country, China's per capita income is still limited, and the marginal impact on people's utility improvement is greater when costs are waived, resulting in significant policy effects. In addition, the influenza vaccination rate in China is very low, providing ample room for the effectiveness of the vaccination policy, which is also one of the reasons why the estimated results in this article are larger than existing conclusions.

This study has some limitations. First, the implementation of the policy is not completely random. Beijing and Shanghai are the most developed cities in China, with development levels even approaching those of developed countries. Based on this, the results obtained in this article differ from existing conclusions. Therefore, for all developing countries, the estimated results in this article may only be a lower bound. In addition, the implementation time of the policy in Beijing and Shanghai is different, which may lead to differences in policy maturity, and the results obtained in this article are only average effects. Last the finding is based on China's policy, which have established a mature supply system for influenza vaccines. However, countries lacking influenza vaccines may not be able to promote this policy.

## 7 Summary

This study provides robust evidence that implementing a free influenza vaccination policy in China can increase vaccination take-up and improve health outcomes. These findings emphasize the importance of government financial subsidies to address the contradiction between the health demands of the older population and budget constraints.

Several policy implications emerge. First, the free provision of basic vaccines is crucial. Preventing diseases through vaccines is the most effective way to solve public health problems, and incentivizing target groups to receive vaccines through price incentives is highly effective in developing countries.

Second, vaccination can bring medium-term non-specific health improvements, so it is necessary to extensively evaluate the benefits of vaccination policy so that valuable vaccination policy cannot be implemented due to the lack of economic benefits.

Finally, the dissemination of information plays a crucial role in improving health, therefore further enhancing the display of health information can enhance the effectiveness of vaccination policy. In addition, widely promoting health information is also a good means to improve health.

## Data Availability

Publicly available datasets were analyzed in this study. This data can be found here: http://cgss.ruc.edu.cn/.

## Footnotes

1 According to survey results by the World Health Organization (WHO) and the United Nations Children's Fund (UNICEF), influenza vaccines were available in 74% of WHO Member States (30% of African countries, 19% of low-income countries), and 66% of WHO Member States (19% of African countries, 12% of low-income countries) had a seasonal influenza vaccination policy in 2022 (6).

2 The information of China's NIP, see Supplementary Materials C.

3 Government document from Beijing Municipal Health Commission. For more details see https://wjw.beijing.gov.cn/xwzx_20031/wnxw/201912/t20191214_1177667.html. The free influenza vaccines mentioned in this paper are uniformly purchased and administered by government departments. The effective ingredients are determined by the CDC based on the situation of the influenza virus in China but do not change over time. In terms of dosage, using the universal standard 15μg vaccine uniformly.

4 Data from The Central People's Government of the People's Republic of China. For more details see https://www.gov.cn/gzdt/2007-10/20/content_780691.htm.

5 Government document from The Central People's Government of the People's Republic of China. For more details see https://www.gov.cn/jrzg/2009-10/13/content_1438437.htm.

6 Unless otherwise specified, all ages are in 2010. In section 3.2, I will provide a detailed explanation of the age processing methods used in this paper.

7 In the study of health effects and further analysis, there may be missing dependent variables in the main sample. Considering the sample size, I don't exclude them directly when constructing the sample.

8 Due to the use of five-category options for frequency-related questions in the questionnaire, some options have a limited number of participants. I transform the original five-category outcomes into binary outcomes, coding “rarely” and “never” as 0, and “always,” “often,” and “sometimes” as 1. The transformation adheres to the following principles: (1) uniform conversion criteria are applied to all indicators; (2) an effort is made to maintain balance among different outcomes.

9 I use the 1% sample of 2010 census data released by the National Bureau of Statistics to make statistics. Only 7% of the people over 60 years old living in Beijing and Shanghai are non-local registered residents.

10 This is consistent with the conclusion drawn from the deworming project implemented in Kenya (47), only free hygiene products can motivate people to use them, while other interventions may fail, and the phenomenon of cost recovery plans leading to a decrease in usage may also apply to China.

11 Multiple choice results show that 48% of the sick people who intentionally did not see a doctor are due to high medical costs.
