# Supplementary material for: Providing free influenza vaccine to increase vaccination take-up and improve physical and mental health: evidence from China
Source: Front Public Health. 2026 Sep 9;14:1883613. doi: 10.3389/fpubh.2026.1883613 (PMC13597904; doi:10.3389/fpubh.2026.1883613)
Supplement: Supplementary file 1 [file Data_Sheet_1.docx]

Providing free influenza vaccine to increase vaccination take-up and improve physical and mental health: Evidence from China

Supplementary Material

Part A. Tables

# 1 Table A1 - Descriptive Statistics – Spouse

| Variable | Mean | Standard deviations |
| --- | --- | --- |
| Influenza vaccination | 0.09 | 0.28 |
| Age | 58.14 | 8.01 |
| Male | 0.44 | 0.50 |
| Education Level |  |  |
| Primary | 0.49 | 0.50 |
| Lower Secondary | 0.28 | 0.45 |
| Upper Secondary | 0.16 | 0.37 |
| Post Secondary | 0.07 | 0.25 |
| Nation: Han | 0.92 | 0.27 |
| Medical Insurance Coverage | 0.92 | 0.27 |
| Household Income |  |  |
| <15K | 0.29 | 0.46 |
| 15K-35K | 0.33 | 0.47 |
| >=35K | 0.28 | 0.45 |
| Missing | 0.10 | 0.30 |
| Suffering from Chronic Diseases | 0.52 | 0.50 |
| Married | 1.00 | 0.00 |
| Number of Children |  |  |
| 0 | 0.00 | 0.07 |
| 1 | 0.30 | 0.46 |
| 2 | 0.31 | 0.46 |
| 3 | 0.23 | 0.42 |
| 4+ | 0.16 | 0.36 |
| Observation | 609 | |
| *Notes:* This table presents descriptive statistics for outcome variables and individual attributes used in spillover analysis. Columns (2) and (3) respectively report the mean and standard deviation of the samples. The sample is selected from the 2010 wave of CGSS data, with the number of 609 observations. Including individuals who are married and have a spouse aged 51-70. Individuals who are the same age as their spouse have been removed, and the situation where both the individual and spouse do not meet policy requirements and meet policy requirements at the same time is not considered. | | |

# 2 Table A2 - Descriptive Statistics - Other Outcome Variables

| Variable | Mean | Standard deviations | N |  |
| --- | --- | --- | --- | --- |
| *Panel A: Other Outcome Variable* |  |  |  |  |
| Self-evaluation of health | 0.43 | 0.50 | 1223 |  |
| Health issues restrict climbing stairs | 0.47 | 0.50 | 1218 |  |
| Health issues restrict the ability to move tables, use vacuum cleaners, play bowling or golf | 0.36 | 0.48 | 1223 |  |
| Affect daily activities due to health | 0.42 | 0.49 | 1220 |  |
| Inability to complete expected work due to health | 0.40 | 0.49 | 1223 |  |
| Feel depressed or discouraged | 0.40 | 0.49 | 1220 |  |
| Feel calm and composed | 0.71 | 0.45 | 1223 |  |
| Feel energetic | 0.54 | 0.50 | 1222 |  |
| Unable to work due to emotions | 0.30 | 0.46 | 1223 |  |
| Feel distracted due to emotions | 0.31 | 0.46 | 1222 |  |
| Smoking multiple times within a month | 0.32 | 0.47 | 1224 |  |
| Drinking multiple times within a month | 0.28 | 0.45 | 1222 |  |
| Visit hospital at least monthly | 0.18 | 0.38 | 1223 |  |
| Accept traditional Chinese medicine treatment | 0.39 | 0.49 | 1224 |  |
| Sick but intentionally not visit doctor | 0.47 | 0.50 | 1115 |  |
| Believe that social is equity | 0.43 | 0.50 | 1219 |  |
| Believe that life is happy | 0.69 | 0.46 | 1222 |  |
| Worry about obtaining medical services | 0.73 | 0.44 | 1219 |  |
| Worry about medical expenses | 0.82 | 0.39 | 1216 |  |
| *Panel B: Alternative Interpretations Variable* |  |  |  |  |
| number of medical and health institutions per 10,000 people | 2.41 | 0.85 | 31 |  |
| number of healthcare personnel per 10,000 people | 49.99 | 15.51 | 31 |  |
| Incidence rate per 100,000 people | 3.23 | 3.67 | 31 |  |
| Mortality rate per 10,000,000 people | 0.02 | 0.08 | 31 |  |
| *Notes:* Panel A of this table presents other outcome variables used in this paper, sourced from the 2010 wave of CGSS data. Panel B of this table presents alternative interpretations variables, where the per capita number of medical and health institutions and per capita number of health personnel are from the National Bureau of Statistics, and disease incidence and mortality data are from the Chinese Center for Disease Control and Prevention. Columns (2) and (3) respectively report the mean and standard deviation of the samples. The number of observations is shown in column (4). | | | | |

# 3 Table A3. Mean of Vaccination Take-up by Age Cohort and Region

|  | Vaccination take-up | | |
| --- | --- | --- | --- |
|  | Beijing Shanghai | Others | Difference |
| *Panel A: Experiment of interest* |  |  |  |
| Aged 61-70 in 2010 | 0.310 | 0.041 | 0.269 |
|  | (0.083) | (0.010) | (0.083) |
| Aged 51-60 in 2010 | 0.075 | 0.068 | 0.007 |
|  | (0.031) | (0.009) | (0.032) |
| Difference | 0.235 | -0.027 | 0.261 |
|  | (0.088) | (0.013) | (0.089) |
| *Panel B: Control Experiment* |  |  |  |
| Aged 51-60 in 2010 | 0.075 | 0.068 | 0.007 |
|  | (0.031) | (0.009) | (0.032) |
| Aged 41-50 in 2010 | 0.108 | 0.094 | 0.013 |
|  | (0.047) | (0.010) | (0.048) |
| Difference | -0.033 | -0.027 | -0.006 |
|  | (0.056) | (0.013) | (0.058) |
| *Notes:* All cells show weighted sample. Standard errors are in parentheses. | | | |

# 4 Table A4. Effect of the Free Influenza Vaccination Policy on Vaccination Take-up - Using different methods to measure alternative interpretations

| Dependent variable |  | Vaccination take-up | | | | | | |
| --- | --- | --- | --- | --- | --- | --- | --- | --- |
|  | (1) | | (2) | (3) | (4) | (5) | (6) | |
| Over 60 years old | 0.229 ^***^ | | -0.021 | 0.188 ^*^ | 0.229 ^***^ | 0.191 ^**^ | 0.209 ^**^ | |
| × Beijing or Shanghai | (0.082) | | (0.029) | (0.097) | (0.082) | (0.076) | (0.086) | |
| Over 60 years old |  | |  | 0.001 |  |  | -0.001 | |
| × Per capital number of beds in medical institutions |  | |  | (0.001) |  |  | (0.003) | |
| Over 60 years old |  | |  |  | -0.025 |  | -0.013 | |
| × Local mortality rate |  | |  |  | (0.112) |  | (0.114) | |
| Over 60 years old |  | |  |  |  | 0.041 | 0.042 | |
| × Pension status |  | |  |  |  | (0.037) | (0.037) | |
| Beijing or Shanghai |  | |  |  |  | 0.051 | 0.050 | |
| × Pension status |  | |  |  |  | (0.036) | (0.036) | |
| Mean of control group | 0.0677 | | 0.117 | 0.0677 | 0.0677 | 0.0677 | 0.0677 | |
| Province FE | √ | | √ | √ | √ | √ | √ | |
| Age FE | √ | | √ | √ | √ | √ | √ | |
| Observations | 1224 | | 1645 | 1224 | 1224 | 1224 | 1224 | |
| *Notes:* All regulations control individual attributes. All regressions employ survey weight for weighting purposes. The SEs reported in parentheses is clustered at the city level. Significance level: *** p < 0.01; ** p < 0.05; * p < 0.1. | | | | | | | |  |

# 5 Table A5. Effect of the Free Influenza Vaccination Policy on Vaccination Take-up - Deleting the individual whose registered residence cannot be determined

| Dependent variable |  | Vaccination take-up | | | | | |
| --- | --- | --- | --- | --- | --- | --- | --- |
|  | (1) | | (2) | (3) | (4) | (5) | (6) |
| Over 60 years old | 0.248 ^**^ | | -0.009 | 0.249 ^**^ | 0.254 ^**^ | 0.239 ^***^ | 0.247 ^***^ |
| × Beijing or Shanghai | (0.100) | | (0.023) | (0.109) | (0.099) | (0.059) | (0.067) |
| Over 60 years old |  | |  | -0.012 |  |  | -0.015 |
| × Per capital number of medical and health institutions |  | |  | (0.023) |  |  | (0.024) |
| Over 60 years old |  | |  |  | 0.004 |  | 0.005 |
| × Local incidence rate |  | |  |  | (0.006) |  | (0.006) |
| Over 60 years old |  | |  |  |  | -0.062 | -0.070 |
| × Working status |  | |  |  |  | (0.042) | (0.044) |
| Beijing or Shanghai |  | |  |  |  | -0.043 | -0.044 |
| × Working status |  | |  |  |  | (0.105) | (0.106) |
| Mean of control group | 0.0827 | | 0.118 | 0.0827 | 0.0827 | 0.0827 | 0.0827 |
| Province FE | √ | | √ | √ | √ | √ | √ |
| Age FE | √ | | √ | √ | √ | √ | √ |
| Observations | 1201 | | 1628 | 1201 | 1201 | 1201 | 1201 |
| *Notes:* All regulations control individual attributes. All regressions employ survey weight for weighting purposes. The SEs reported in parentheses is clustered at the city level. Significance level: *** p < 0.01; ** p < 0.05; * p < 0.1. | | | | | | | |

# 6 Table A6. Effect of the Free Influenza Vaccination Policy on Vaccination Take-up - Using the 2021 wave of CGSS data

| Dependent variable |  | Vaccination take-up | | | | | | |
| --- | --- | --- | --- | --- | --- | --- | --- | --- |
|  | (1) | | (2) | (3) | (4) | (5) | (6) | |
| Over 60 years old | 0.117 ^***^ | | -0.038 | 0.137 ^***^ | 0.120 ^**^ | 0.098 ^***^ | 0.103 ^*^ | |
| × Beijing or Shanghai | (0.020) | | (0.022) | (0.019) | (0.053) | (0.021) | (0.052) | |
| Over 60 years old |  | |  | 0.007 |  |  | 0.006 | |
| × Per capital number of medical and health institutions |  | |  | (0.005) |  |  | (0.004) | |
| Over 60 years old |  | |  |  | -0.000 |  | 0.000 | |
| × Local incidence rate |  | |  |  | (0.000) |  | (0.000) | |
| Over 60 years old |  | |  |  |  | 0.051 | 0.051 | |
| × Working status |  | |  |  |  | (0.059) | (0.058) | |
| Beijing or Shanghai |  | |  |  |  | -0.072 ^***^ | -0.070 ^**^ | |
| × Working status |  | |  |  |  | (0.025) | (0.025) | |
| Mean of control group | 0.0518 | | 0.0743 | 0.0518 | 0.0518 | 0.0518 | 0.0518 | |
| Province FE | √ | | √ | √ | √ | √ | √ | |
| Age FE | √ | | √ | √ | √ | √ | √ | |
| Observations | 1072 | | 944 | 1072 | 1072 | 1072 | 1072 | |
| *Notes:* All regulations control individual attributes. All regressions employ survey weight for weighting purposes. The SEs reported in parentheses is clustered at the city level. Significance level: *** p < 0.01; ** p < 0.05; * p < 0.1. | | | | | | | |  |

# 7 Table A7. Effect of the Free Influenza Vaccination Policy on Vaccination Take-up – Coefficients of the interactions between dummies indicating age in 2010 and the dummies of Beijing or Shanghai

| Dependent variable | Vaccination take-up | |
| --- | --- | --- |
| Age in 2010 | Coefficient  (1) | S.E.  (2) |
| 51 | 0.151 | (0.127) |
| 52 | 0.085 | (0.115) |
| 53 | 0.297 ^**^ | (0.127) |
| 54 | 0.021 | (0.105) |
| 55 | 0.143 | (0.151) |
| 56 | 0.236 ^**^ | (0.117) |
| 57 | 0.133 | (0.108) |
| 58 | 0.154 | (0.106) |
| 59 | 0.015 | (0.149) |
| 61 | 0.309 ^**^ | (0.119) |
| 62 | 0.454 ^***^ | (0.152) |
| 63 | 0.705 ^**^ | (0.277) |
| 64 | 0.134 | (0.107) |
| 65 | 0.283 ^*^ | (0.147) |
| 66 | 0.721 ^***^ | (0.162) |
| 67 | 0.312 | (0.262) |
| 68 | 0.454 | (0.288) |
| 69 | 0.097 | (0.152) |
| 70 | 0.507 | (0.338) |
| Mean of control group | 0 | |
| Province FE | √ | |
| Age FE | √ | |
| Observations | 1224 | |
| *Notes:* All regulations control individual attributes. All regressions employ survey weight for weighting purposes. The SEs reported in parentheses is clustered at the city level. Significance level: *** p < 0.01; ** p < 0.05; * p < 0.1. | | |

# 8 Table A8. Generalized Synthetic Control Method – Average of Average Treatment Effect on the Treated

|  | Estimate | S.E. | CI.lower | CI.upper | p.value |  |
| --- | --- | --- | --- | --- | --- | --- |
| ATT.avg | 0.2598 | 0.0790 | 0.1050 | 0.4145 | 0.0010 |  |
| Province FE | √ | | | | |  |
| Age FE | √ | | | | |  |
| Observations | 478 | | | | |  |
| Treated Provinces | 2 | | | | |  |
| Control Provinces | 29 | | | | |  |
| *Notes:* This table presents the average of average treatment effect on the treated estimated by the generalized synthetic control method, including 2 treated groups and 29 control groups, with the number of 478 observations. Among them, age and province fixed effects are controlled, and the standard error are based on parameter bootstraps of 1,000 times. | | | | | | |

# 9 Table A9. Generalized Synthetic Control Method – Average Treatment Effect on the Treated by Age

| Age in 2010 | ATT | S.E. | CI.lower | CI.upper | p.value |
| --- | --- | --- | --- | --- | --- |
| 51 | -0.0289 | 0.0936 | -0.2124 | 0.1545 | 0.7571 |
| 52 | 0.0129 | 0.1144 | -0.2114 | 0.2371 | 0.9106 |
| 53 | 0.2186 | 0.0958 | 0.0309 | 0.4062 | 0.0225 |
| 54 | -0.0103 | 0.0510 | -0.1103 | 0.0897 | 0.8394 |
| 55 | -0.0161 | 0.0811 | -0.1750 | 0.1428 | 0.8425 |
| 56 | -0.0493 | 0.0927 | -0.2310 | 0.1323 | 0.5945 |
| 57 | -0.0430 | 0.0764 | -0.1928 | 0.1068 | 0.5737 |
| 58 | -0.1395 | 0.1042 | -0.3438 | 0.0648 | 0.1808 |
| 59 | -0.0255 | 0.1198 | -0.2603 | 0.2092 | 0.8312 |
| 60 | 0.0340 | 0.1394 | -0.2393 | 0.3073 | 0.8072 |
| 61 | 0.1010 | 0.0752 | -0.0465 | 0.2485 | 0.1794 |
| 62 | 0.3888 | 0.1067 | 0.1797 | 0.5979 | 0.0003 |
| 63 | 0.5788 | 0.0704 | 0.4409 | 0.7168 | 0.0000 |
| 64 | -0.1100 | 0.3343 | -0.7652 | 0.5452 | 0.7421 |
| 65 | 0.3472 | 0.1189 | 0.1143 | 0.5802 | 0.0035 |
| 66 | 0.6543 | 0.2659 | 0.1331 | 1.1754 | 0.0139 |
| 67 | 0.3534 | 0.1484 | 0.0625 | 0.6443 | 0.0173 |
| 68 | 0.6382 | 0.5023 | -0.3463 | 1.6227 | 0.2039 |
| 69 | -0.0894 | 0.3091 | -0.6953 | 0.5165 | 0.7723 |
| 70 | 0.3762 | 0.0775 | 0.2243 | 0.5281 | 0.0000 |
| Province FE | √ | | | | |
| Age FE | √ | | | | |
| Observations | 478 | | | | |
| Treated Provinces | 2 | | | | |
| Control Provinces | 29 | | | | |
| *Notes:* This table presents the average treatment effect on the treated by period estimated by the generalized synthetic control method, including 2 treated groups and 29 control groups, with the number of 478 observations. Among them, age and province fixed effects are controlled, and the standard errors are based on parameter bootstraps of 1,000 times. | | | | | |

# 10 Table A10. Heterogeneous effect of the Free Influenza Vaccination Policy on Vaccination Take-up

| Dependent variable | Vaccination take-up | | | | | | | |  |
| --- | --- | --- | --- | --- | --- | --- | --- | --- | --- |
|  | (1) | (2) | (3) | (4) | (5) | (6) | (7) |  |  |
| Over 60 years old | 0.355 ^**^ | 0.171 ^***^ | -0.015 | 0.305 ^***^ | 0.482 ^**^ | 0.118 ^**^ | 0.213 |  |  |
| × Beijing or Shanghai | (0.170) | (0.055) | (0.071) | (0.052) | (0.238) | (0.058) | (0.136) |  |  |
| Mean of control group | 0.0594 | 0.073 | 0.0564 | 0.0853 | 0 | 0.0454 | 0.0951 |  |  |
| Province FE | √ | √ | √ | √ | √ | √ | √ |  |  |
| Age FE | √ | √ | √ | √ | √ | √ | √ |  |  |
| Observations | 592 | 632 | 571 | 653 | 619 | 318 | 287 |  |  |
|  | (8) | (9) | (10) | (11) |  |  |  |  |  |
| Over 60 years old | 0.172 ^***^ | 0.271 ^**^ | 0.512 ^**^ | 0.105 |  |  |  |  |  |
| × Beijing or Shanghai | (0.056) | (0.116) | (0.216) | (0.096) |  |  |  |  |  |
| Mean of control group | 0.179 | 0.017 | 0.024 | 0.0914 |  |  |  |  |  |
| Province FE | √ | √ | √ | √ |  |  |  |  |  |
| Age FE | √ | √ | √ | √ |  |  |  |  |  |
| Observations | 521 | 703 | 186 | 305 |  |  |  |  |  |
| *Notes:* All regulations control individual attributes. All regressions employ survey weight for weighting purposes. The SEs reported in parentheses is clustered at the city level. Significance level: *** p < 0.01; ** p < 0.05; * p < 0.1. | | | | | | | | | |

Part B. Figures

# 11 Figure B1. Result of Random Placebo Test

Note: This figure presents the results obtained by randomly assigning policy implementation labels to different regions and repeatedly estimating the basic specifications of this paper 1000 times. The X-axis in the figure represents the estimated coefficients, and the Y-axis represents the corresponding density. The black line shows the effect results of the false treatment group estimation, while the red line represents the true estimation results. In the 1000 false treatment group estimates, there is no case where the coefficient is greater than the true coefficient, and there is not enough reason to believe that the true results are due to accidental factors.

# 12 Figure B2 - Generalized Synthetic Control Method – Average Treatment Effect on the Treated by Period

Notes: This figure presents the average treatment effect on treated of the free influenza vaccination policy on vaccination take-up by different ages estimated using the generalized synthetic control method. The solid line represents the estimated coefficient, while the dashed line represents the 95% confidence interval. The results show that there is no significant difference in vaccination take-up between the treatment group and the synthesized control group when the age does not meet policy requirements. However, when the age meets the policy requirements, the vaccination take-up of the treatment group is significantly higher than the control group. Proving the credibility of the existing results. The standard errors are based on parameter bootstraps of 1,000 times. Table A8 of the Supplementary Materials presents these results in table format.

# 13 Figure B3. Heterogeneous Effect of the Free Influenza Vaccination on Vaccination Take-up

Note: The middle column of the figure shows the estimation results of the coefficients, the dashed line represents the baseline results, the red dots represent the statistical significance at the 5% level, and the horizontal line represents the 95% confidence interval. The right column displays the specific numerical values of the estimation results and the percentage change relative to the control group. When the mean of the control group is 0, it is displayed as Inf. Supplementary Materials Table A10 presents these results in table format.

Part C. Policy informations

# 14 China’s NIP

China’s NIP was established and implemented in 1978, with the first batch including 4 vaccines that can prevent a total of 6 diseases.^[1]^ In 2002 and 2007, the NIP was expanded to include 14 types of free vaccines to prevent 15 corresponding diseases.^[2]^ The target audience for all vaccines in the NIP is children, who are vaccinated according to the immunization procedures provided by the Chinese Center for Disease Control and Prevention (CDC). Currently, the vaccination rate of vaccines in the immunization program has reached over 99%.^[3]^ For the over-age group, they can voluntarily choose to receive supplementary vaccines within the NIP without paying any fees.

1. The four vaccines include BCG, OPV, DTaP, and MV to prevent tuberculosis, poliomyelitis, diphtheria, pertussis, tetanus, and measles.
2. In 2002, the hepatitis B vaccine was incorporated into the NIP. In 2007, rubella, mumps, Japanese encephalitis, epidemic cerebrospinal meningitis, and hepatitis A were added to the protection under the NIP. Additionally, vaccines for epidemic hemorrhagic fever, anthrax, and leptospirosis were made available for specific populations. For more details see https://www.gov.cn/gzdt/2008-02/19/content_893572.htm
3. This data is an estimated value, sourced from the WHO website. For more details see https://platform.who.int/data/
